# Outcomes of subsequent pregnancy following obstetric transfusion in a first birth

**DOI:** 10.1371/journal.pone.0203195

**Published:** 2018-09-28

**Authors:** Jillian A. Patterson, Tanya Nippita, Deborah A. Randall, David O. Irving, Jane B. Ford, for the Obstetric Transfusion Steering Group

**Affiliations:** 1 Clinical and Population Perinatal Health Research, Kolling Institute, Northern Sydney Local Health District, St Leonards, New South Wales, Australia; 2 Northern Clinical School, The University of Sydney, St Leonards, Australia; 3 Department of Obstetrics and Gynaecology, Royal North Shore Hospital, Northern Sydney Local Health District, St Leonards, New South Wales, Australia; 4 Research and Development, Australian Red Cross Blood Service, Sydney, New South Wales, Australia; King’s College London, UNITED KINGDOM

## Abstract

**Background:**

Increasing rates of postpartum haemorrhage and obstetric transfusion mean that more women are entering subsequent pregnancies with a history of blood transfusion. This study investigates subsequent pregnancy outcomes of women with a prior obstetric red cell transfusion, compared to women without a transfusion.

**Methods:**

All women with a first pregnancy resulting in a liveborn singleton infant of at least 20 weeks gestation delivering in hospitals in New South Wales, Australia, between 2003 and 2012 were included in the study, with followup for second births until June 2015. Linked hospital and births data were used to identify women with a transfusion and/or postpartum haemorrhage in their first birth, time to second pregnancy and adverse birth outcomes (including transfusion, postpartum haemorrhage and severe morbidity) in their subsequent birth.

**Results:**

There were 358,384 singleton births to primiparous women, with 1.4% receiving an obstetric blood transfusion. Sixty-three percent of women had at least one subsequent birth. The relative risk (RR) of requiring a transfusion in a second birth was 4.9 (95% CI 4.1,6.1) for women with a previous transfusion compared with women without. The risk (RR) of severe morbidity in a second birth was 4.1 times higher (95% CI 2.2,7.4) for those receiving a transfusion without haemorrhage in their first birth compared with women with neither haemorrhage nor transfusion.

**Conclusion:**

It is important to consider a woman’s history of transfusion and/or haemorrhage as part of her obstetric history to ensure management in a manner that minimises risk in subsequent pregnancies.

## Introduction

Rates of excessive bleeding post-childbirth (postpartum haemorrhage, PPH) and obstetric transfusion continue to rise.[1–4] One consequence of increasing haemorrhage and transfusion rates is that more women are entering pregnancy with a history of haemorrhage and/or transfusion from their previous birth. Studies have reported that women with a PPH in their first birth have around 3 times the risk of PPH in their second birth compared with a woman with no history of PPH.[5,6] Few studies have described the recurrence risk of obstetric transfusion due to low numbers of cases (1–2% of births), instead using it as an indicator of more severe haemorrhage. [5, 6] One study, from Denmark, however found previous transfusion a strong predictor of the need for transfusion following subsequent births, however did not specifically consider transfusion with or without haemorrhage. [7]

Although it is difficult to predict which women will experience postpartum haemorrhage, risk factors for haemorrhage are an important consideration in planning the appropriate place of birth in subsequent pregnancies. As much of the initial decision making around place of birth occurs early in pregnancy, there is benefit to both clinician and the woman in assessing a future risk of haemorrhage based on factors known from the woman’s prior pregnancy.

While a number of studies have investigated recurrence risk of haemorrhage in a subsequent pregnancy, few studies have also considered other outcomes including the likelihood of subsequent pregnancy and the development of other complications (eg morbidity, transfusion). Although reasons for recurrent haemorrhage are not fully understood, recurrent haemorrhage may occur due to recurrence of predisposing factors (abnormal placentation, hypertension etc). [6] Routinely collected health data, such as those gathered in hospital administrative databases and birth records, are useful sources of data to investigate recurrence risk and rare outcomes, as they provide large sample sizes and the ability to follow women and their health outcomes through subsequent pregnancies.

This study aims to use routinely collected data to determine (1) the likelihood of a second birth following blood transfusion at the first birth, (2) to examine pregnancy outcomes in a second birth among women who received a blood transfusion in their first birth and (3) to identify the relative importance of transfusion compared to other first birth factors in a woman’s risk for complications in her second pregnancy.

## Materials and methods

The study population included all primiparous women delivering a liveborn singleton infant of at least 20 weeks gestation in New South Wales (NSW) hospitals between 2003 and 2012 (‘index birth’). Women with bleeding or platelet disorders were excluded. Women were followed until 30 June 2015 (minimum 2.5 years followup) to identify subsequent (second) birth of one or more infants >20 weeks. Women with incomplete pregnancy history (for example only having first and third birth records) were excluded.

Maternal and pregnancy characteristics were available from the NSW Perinatal Data Collection (‘birth data’), which is a statutory collection of data on all births of at least 20 weeks gestation or 400 grams birthweight in NSW. The Admitted Patients Data Collection (‘hospital data’) contains data on all inpatient hospital admissions in NSW with diagnoses and procedures coded according to the International Classification of Diseases version 10 –Australian Modification, and the Australian Classification of Health Interventions respectively. The hospital data were used to obtain information on medical conditions (including PPH defined as blood loss >500mL after vaginal birth or 750mL after caesarean birth), red blood cell transfusion and other procedures. The exposure of interest was blood cell transfusion occurring at any time during the first pregnancy (antenatally, during the birth or postnatal admissions up to six weeks after birth). This was further divided by whether or not the woman had experienced a PPH. Transfusion is reliably recorded in the hospital data (sensitivity 83.1 95%CI(52.2,97.7), specificity 99.9 95%CI(99.7,100)), and postpartum haemorrhage has some underreporting (Sn 73.8% (95%CI(63.1,82.8), Sp 98.9 95%CI(98.1,99.4)). [8] The birth and hospital data were probabilistically linked by the NSW Centre for Health Record Linkage, and datasets stripped of personal identifiers were supplied to the researchers.

The primary outcomes were interpregnancy interval (time between first birth and conception of second pregnancy based on gestational age at second birth), red blood cell transfusion (at any time during the second pregnancy/postnatal period), PPH at the second birth and severe maternal morbidity at the second birth. The specific timing of the transfusion relative to the delivery for each pregnancy is not available in the data. Severe maternal morbidity was measured using a validated composite indicator of diagnoses and procedures indicative of an adverse outcome, including mechanical ventilation, dialysis, cardiac arrest, cerebro-vascular accident, obstetric embolism and shock. [9] The indicator as originally defined includes transfusion, however this component was removed and assessed separately. Pregnancy losses prior to 20 weeks gestation were identified from hospital diagnoses of miscarriage occurring between the end of the first pregnancy and (where applicable) prior to conception of the second birth.

As PPH is known to be under-reported, a sensitivity analysis was conducted where conditions commonly associated with PPH (3^rd^/4^th^ degree tears and obstetric trauma, severe delivery and postnatal complications, intrapartum haemorrhage and placental abruption) were classified as PPH.

Trends over time were assessed using the Cochran Armitage test. Kaplan Meier curves and log rank tests were used to compare time to second pregnancy between study groups. Among women with no second births recorded before 30 June 2015 interpregnancy intervals were censored at 1 September 2014. Modified Poisson regression with robust error variances was used to examine the relationship between first birth characteristics and: (1) transfusion, (2) PPH, and (3) severe maternal morbidity at the second birth. This method adjusts for potential confounding factors, producing adjusted relative risks (aRR), which are directly interpreted as the rate of the outcome in one group relative to the other group. In order to determine the relative importance of first birth factors on second birth outcomes, transfusion with or without haemorrhage (the primary exposure) and non-modifiable first birth factors such as women’s characteristics or complications (eg chronic hypertension, morbidly adherent placenta, placenta praevia) that are likely to also be present in a second birth were included in the models. Management factors (including mode of birth and induction of labour) were not included in modelling as they likely lie on the causal pathway between various medical conditions and adverse events.[10] Although first birth management may influence morbidity at a second birth (such as repeat caesarean section), including them in the model would bias estimates of the maternal conditions which they were used to treat, and which were of interest in our study. Gestational age and small for gestational age were not considered causally related to transfusion, as these can be related to the indication for transfusion (eg praevia, placenta accreta). [11] Factors to be considered in the model were identified from the literature as factors related to both risk of morbidity and postpartum haemorrhage/transfusion. [3, 7, 12–14] These were private insurance status, Australian country of birth, maternal smoking, artificial reproductive technology use, pregnancy hypertension, chronic hypertension, gestational diabetes, chronic conditions, large for gestational age (>90^th^ centile), morbidly adherent placenta, maternal age, socioeconomic status (quintiles), year of birth and PPH type identified at the first birth, with categories as shown in Table 1. All factors were entered into and retained in the model. Maternal age was considered in three categories: Under 20 years, 20–34 years and 35 and over, reflecting the difference in risks between teenage and older mothers. Chronic medical conditions included chronic renal disease, cardiac conditions, chronic obstructive pulmonary disease, psychiatric disorders and thyroid and autoimmune conditions. [15]

**Table 1:** Maternal and pregnancy characteristics (at the first birth) of women receiving and not receiving a transfusion.

Variable	+RBC^b^ –PPH^c^*	+RBC+ PPH	-RBC + PPH	- RBC—PPH
	% (95% CI)	% (95% CI)	% (95% CI)	% (95% CI)
Total (N)	949	4176	27313	325946
Maternal Age				
Under 20	11.0 (9.0, 12.9)	8.7 (7.8, 9.5)	6.7 (6.4, 7.0)	7.2 (7.1, 7.2)
20–34	72.8 (70.0, 75.6)	77.2 (75.9, 78.5)	79.7 (79.3, 80.2)	78.4 (78.3, 78.6)
35+	16.2 (13.9, 18.6)	14.1 (13.1, 15.2)	13.6 (13.2, 14.0)	14.4 (14.3, 14.5)
Private Insurance	28.1 (25.3, 31.0)	24.2 (22.9, 25.5)	25.9 (25.4, 26.4)	37.8 (37.7, 38.0)
Smoker	11.3 (9.3, 13.3)	8.7 (7.9, 9.6)	8.6 (8.3, 9.0)	10.3 (10.2, 10.4)
ART^a^ use	7.0 (5.3, 8.6)	4.8 (4.2, 5.5)	4.0 (3.8, 4.2)	4.4 (4.3, 4.5)
Pregnancy hypertension	18.3 (15.9, 20.8)	16.3 (15.1, 17.4)	12.4 (12.0, 12.8)	10.8 (10.6, 10.9)
Chronic hypertension	1.7 (0.9, 2.5)	1.1 (0.8, 1.4)	1.0 (0.8, 1.1)	0.8 (0.8, 0.9)
Gestational diabetes	6.3 (4.8, 7.9)	6.5 (5.8, 7.3)	6.3 (6.0, 6.6)	6.0 (5.9, 6.0)
Pre-existing diabetes	0.9 (0.3, 1.6)	0.3 (0.1, 0.4)	0.4 (0.3, 0.4)	0.4 (0.4, 0.5)
Chronic conditions	8.1 (6.4, 9.9)	3.8 (3.2, 4.4)	2.9 (2.7, 3.1)	2.6 (2.5, 2.7)
Severe maternal morbidity	14.1 (11.9, 16.3)	8.9 (8.0, 9.8)	1.2 (1.0, 1.3)	0.4 (0.4, 0.4)
Gestational Age				
20–32 weeks	5.6 (4.1, 7.0)	1.3 (1.0, 1.7)	1.0 (0.9, 1.1)	1.1 (1.1, 1.1)
33–36 weeks	11.4 (9.4, 13.4)	4.2 (3.6, 4.8)	3.6 (3.4, 3.8)	4.9 (4.8, 4.9)
37+ weeks	83.0 (80.6, 85.4)	94.4 (93.7, 95.1)	95.4 (95.1, 95.6)	94.0 (93.9, 94.1)
Mode of birth				
Normal vaginal delivery	16.8 (14.4, 19.1)	41.6 (40.1, 43.1)	48.7 (48.1, 49.3)	47.9 (47.7, 48.0)
Pre-labour Caesarean	24.3 (21.6, 27.1)	5.0 (4.3, 5.6)	4.8 (4.5, 5.0)	11.3 (11.2, 11.4)
Intrapartum Caesarean	32.9 (29.9, 35.9)	14.8 (13.7, 15.9)	13.8 (13.4, 14.3)	20.4 (20.3, 20.6)
Instrumental delivery	25.4 (22.6, 28.2)	36.6 (35.1, 38.1)	31.3 (30.7, 31.8)	19.9 (19.8, 20.0)
Induction of labour	34.4 (31.3, 37.4)	42.7 (41.2, 44.2)	39.5 (38.9, 40.0)	32.4 (32.3, 32.6)
Birthweight				
<10^th^ centile	11.3 (9.3, 13.3)	6.7 (5.9, 7.4)	7.5 (7.2, 7.8)	12.2 (12.1, 12.4)
10–90 centile	75.3 (72.6, 78.1)	78.6 (77.3, 79.8)	80.6 (80.1, 81.1)	80.6 (80.4, 80.7)
>90^th^ centile	13.4 (11.2, 15.5)	14.8 (13.7, 15.9)	11.9 (11.5, 12.2)	7.2 (7.1, 7.3)
Hospital level				
Tertiary	37.1 (34.0, 40.2)	36.2 (34.7, 37.6)	37.9 (37.4, 38.5)	29.6 (29.4, 29.7)
Other Urban	25.5 (22.7, 28.3)	29.4 (28.0, 30.8)	29.5 (29.0, 30.1)	25.0 (24.8, 25.1)
Regional	24.0 (21.3, 26.7)	21.1 (19.9, 22.4)	17.6 (17.2, 18.1)	18.8 (18.7, 18.9)
Private	13.4 (11.2, 15.5)	13.2 (12.2, 14.3)	14.9 (14.5, 15.3)	26.6 (26.5, 26.8)
Second pregnancy	51.7 (48.6, 54.9)	57.4 (55.9, 58.9)	61.8 (61.2, 62.3)	63.7 (63.5, 63.8)
Pregnancy loss	9.5 (7.6, 11.3)	8.6 (7.7, 9.4)	8.2 (7.8, 8.5)	8.1 (8.0, 8.2)

^a^ART, Assisted reproductive technology;

^b^RBC, Red blood cell;

^c^PPH, Postpartum haemorrhage.

* +RBC +PPH: Transfusion and haemorrhage; +RBC-PPH: Transfusion without haemorrhage; -RBC +PPH: Haemorrhage without transfusion;-RBC–PPH: neither haemorrhage nor transfusion.

All analysis was conducted in SAS v 9.4. Ethics approval was obtained from the NSW Population and Health Services Research Ethics Committee.

## Results

### First births

Between 2003 and 2012 there were 358,384 singleton births to primiparous women. Of these 5125 (1.4%) received a blood transfusion during their pregnancy, birth or the postnatal period, with the rate increasing from 1.0% in 2003 to 1.7% in 2012 (p<0.0001). The postpartum haemorrhage rate in primipara increased from 7.1% to 10.2% (p<0.0001) in the same period. Those receiving transfusions in their first pregnancy were more likely to have had hypertension or a chronic medical condition, and to give birth preterm, and were less likely to have private insurance (Table 1) and these differences tended to be more pronounced where the transfusion was given in the absence of haemorrhage. Of women who received a blood transfusion in their first pregnancy but were not reported to have experienced a PPH, 21.8% had another bleeding or placental issue (including antepartum haemorrhage, morbidly adherent placenta, placenta praevia), 3.6% had severe delivery or postnatal complications, 12.2% had obstetric trauma and 56.9% had a record of anaemia in the first pregnancy.

### Determining the likelihood of a second birth following blood transfusion at the first birth

**Fig 1:**
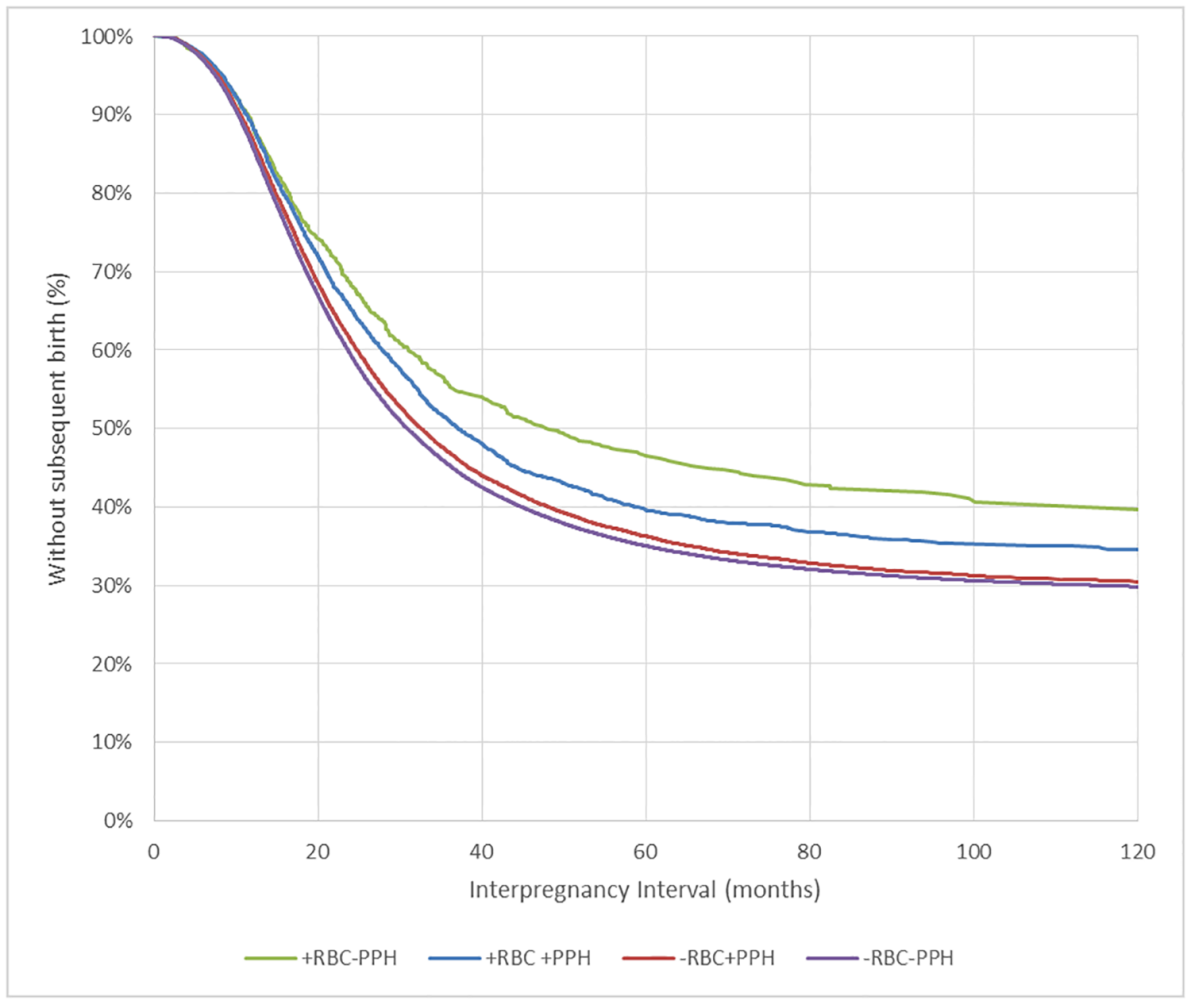
Interpregnancy interval for women following transfusion and/or postpartum haemorrhage in their first birth. RBC = Blood transfusion; PPH = postpartum haemorrhage.

With followup until June 2015, 63% of women had a subsequent birth conceived before 1 September 2014, with a median interpregnancy interval of 31.2 months (95%CI 31.0,31.3). The interpregnancy interval was longest in women receiving a transfusion in the absence of haemorrhage in the index pregnancy (48.4 months 95%CI(41.3,60.1) followed by those who received a transfusion and experienced haemorrhage (37.1, 95%CI(35.3,39.6), those with haemorrhage but no transfusion (32.6, 95%CI(32.0,33.3) and those with neither haemorrhage nor transfusion (30.9 95%CI(30.8,31.1) (Fig 1).

### Pregnancy outcomes in a second birth

Of the 227,247 women who had a first and second birth recorded in the dataset, 2890 (1.3%) had a transfusion in their first pregnancy and 2153 (1.0%) in their second pregnancy, and there was a recurrence risk of 4.9 (95%CI 4.1,6.1). Of women receiving a transfusion in their first birth, 571 (19.8%) experienced a postpartum haemorrhage in their second pregnancy, with 127 (4.4%) also receiving a subsequent transfusion. Of the 19,268 (8.5%) women with a PPH in their first pregnancy, 3319 (17.2%) had a PPH in their second pregnancy, giving a recurrence risk of 2.8 (95%CI 2.7,2.9).

Severe maternal morbidity in the second birth was highest (2.2%) for those who received a transfusion without haemorrhage in their index birth (Table 2). Women receiving a blood transfusion in their first birth (with (1.0%) or without a diagnosis of haemorrhage (1.1%)) were more likely to have a diagnosis of isoimmunisation recorded in hospital records for their second pregnancy compared with those not receiving a transfusion (0.4%).

**Table 2:** Second birth outcomes associated with transfusion and/or postpartum haemorrhage in a first birth (Univariate analysis).

	First birth exposures			
Second birth Outcomes	+RBC–PPH*	+RBC+ PPH	-RBC + PPH	- RBC—PPH
	% (95% CI)	% (95% CI)	% (95% CI)	% (95% CI)
Total	491	2399	16869	207488
Severe Maternal morbidity	2.2 (0.9, 3.5)	1.0 (0.6, 1.4)	0.6 (0.5, 0.7)	0.5 (0.4, 0.5)
Isoimmunisation	1.0 (0.1, 1.9)	1.1 (0.7, 1.5)	0.4 (0.3, 0.5)	0.4 (0.4, 0.5)
Postpartum Haemorrhage	11.8 (9.0, 14.7)	21.4 (19.7, 23.0)	16.6 (16.1, 17.2)	6.2 (6.1, 6.3)
Red Blood Cell transfusion	3.3 (1.7, 4.8)	4.6 (3.8, 5.5)	1.9 (1.7, 2.2)	0.8 (0.8, 0.9)
Preterm birth	7.7 (5.4, 10.1)	5.5 (4.6, 6.4)	4.7 (4.4, 5.0)	5.0 (5.0, 5.1)
Pregnancy loss (prior to second birth)	13.6 (10.6, 16.7)	17.0 (15.5, 18.5)	14.6 (14.1, 15.2)	14.0 (13.9, 14.2)
Change to higher level hospital	10.6 (7.9, 13.3)	8.6 (7.5, 9.8)	8.4 (8.0, 8.8)	8.3 (8.2, 8.5)
Hospital change- Public to Private	8.8 (6.3, 11.3)	7.6 (6.5, 8.6)	6.5 (6.1, 6.9)	4.7 (4.6, 4.8)
Total regional births	24.8 (21.0, 28.7)	23.3 (21.6, 25.0)	17.6 (17.0, 18.2)	19.2 (19.0, 19.3)
Change to higher level hospital (% of regional births)	5.1 (3.1, 7.0)	3.2 (2.5, 3.9)	2.3 (2.1, 2.5)	2.5 (2.4, 2.6)

* +RBC +PPH: Transfusion and haemorrhage; +RBC-PPH: Transfusion without haemorrhage; -RBC +PPH: Haemorrhage without transfusion;-RBC–PPH: neither haemorrhage nor transfusion.

Women experiencing either haemorrhage or transfusion at a public hospital for their first birth were more likely to change to a private hospital for their second birth compared with women with uncomplicated first deliveries (Table 2).

### Relative importance of transfusion in a woman’s risk for complications in a second birth

After adjustment, women who received a transfusion in their first pregnancy were more likely to experience **severe morbidity** in their second birth, with women receiving transfusion other than in the context of PPH at higher risk (adjusted relative risk (aRR) 4.1 (2.2,7.4)) than women receiving a transfusion in the context of PPH (aRR 2.0 (1.3,3.0)) or those with PPH alone (aRR 1.3 (1.0,1.6)) (Table 3). Chronic medical conditions and placenta praevia also increased the risk of severe maternal outcomes at the following birth to a similar extent to transfusion in the context of PPH. Mothers who were aged 35 or older at their first pregnancy were at increased risk of severe morbidity in their second pregnancy (aRR 1.5 (1.2,1.8)), and both younger and older mothers tended to be at increased risk of postpartum haemorrhage or transfusion in their second delivery. A sensitivity analysis reclassifying 12,568 women who likely had an unreported PPH (N = 12400 (3.8%) with and N = 168 (17.7%) without transfusion) as having had a PPH showed a similar pattern (data not shown).

**Table 3:** First pregnancy factors associated with increased risk of severe maternal morbidity in a second birth, amongst women with a first and second birth.

		Severe maternal morbidty n(%)	aRR^e^	95% Confidence Interval	p- value
Private Insurance	Yes	408/ 87759 (0.5)	0.9	(0.8, 1.0)	0.2
	No	673/ 139488 (0.5)	Ref ^f^		
Australian born	Yes	779/ 162402 (0.5)	1	(0.9, 1.2)	0.7
	No	302/ 64845 (0.5)	Ref		
Smoker	Yes	108/ 21348 (0.5)	1.1	(0.9, 1.4)	0.3
	No	970/ 205290 (0.5)	Ref		
Any ART^a^	Yes	50/ 8205 (0.6)	1.1	(0.8, 1.5)	0.5
	No	1031/ 219042 (0.5)	Ref		
Pregnancy hypertension	Yes	157/ 24472 (0.6)	1.3	(1.1, 1.6)	<0.01
	No	924/ 202775 (0.5)	Ref		
Chronic hypertension	Yes	16/ 1674 (1.0)	1.5	(0.9, 2.5)	0.2
	No	1065/ 225573 (0.5)	Ref		
Gestational diabetes	Yes	67/ 11646 (0.6)	1.2	(0.9, 1.5)	0.3
	No	1014/ 215601 (0.5)	Ref		
Chronic conditions	Yes	76/ 5610 (1.4)	2.8	(2.2, 3.6)	< .0001
	No	1005/ 221637 (0.5)	Ref		
Large for gestational age	Yes	110/ 17495 (0.6)	1.3	(1.1, 1.6)	0.02
	No	971/ 209752 (0.5)	Ref		
Morbidly adherent placenta	Yes	5/ 428 (1.2)	1.8	(0.8, 4.3)	0.3
	No	1076/ 226819 (0.5)	Ref		
Placenta praevia	Yes	23/ 1644 (1.4)	2.5	(1.6, 3.8)	<0.01
	No	1058/ 225603 (0.5)	Ref		
Maternal age	Under 20	66/ 16631 (0.4)	0.8	(0.6, 1.1)	<0.01
	20–34	862/ 188582 (0.5)	Ref		
	35+	153/ 21999 (0.7)	1.5	(1.2, 1.8)	
Socioeconomic status	Highest disadvantage	199/ 44950 (0.4)	0.9	(0.8, 1.1)	0.2
	2	181/ 42651 (0.4)	0.9	(0.7, 1.1)	
	3	229/ 42426 (0.5)	1.1	(0.9, 1.3)	
	4	218/ 44902 (0.5)	1.0	(0.8, 1.2)	
	Highest SES^c^	247/ 50191 (0.5)	Ref		
PPH^b^ Type**	+PPH+RBC^d^	24/ 2399 (1.0)	2.0	(1.3, 3.0)	<0.01
	+PPH–RBC	103/ 16869 (0.6)	1.3	(1.0, 1.6)	
	-PPH+RBC	11/ 491 (2.2)	4.1	(2.2, 7.4)	
	-PPH-RBC	943/ 207488 (0.5)	Ref		
Year of birth			1	(1.0, 1.0)	0.2

^a^ART: Assisted reproductive technology;

^b^PPH: Postpartum haemorrhage;

^c^SES: socioeconomic status;

^d^RBC, Red blood cell;,

^**e**^aRR: Adjusted Relative Risk.

^f^Ref: reference category

** +RBC +PPH: Transfusion and haemorrhage; +RBC-PPH: Transfusion without haemorrhage; -RBC +PPH: Haemorrhage without transfusion;-RBC–PPH: neither haemorrhage nor transfusion.

When considering **PPH at the second birth**, PPH with transfusion at the first birth was associated with a threefold increase in risk (aRR 3.0 (2.8,3.3)), while PPH with no transfusion (aRR 2.4 (2.4–2.5)) and transfusion with no PPH (aRR 1.7 (1.3,2.2)) were associated with a lower degree of increased risk (Table 4). These factors were stronger predictors of PPH than other first pregnancy factors. Women with a transfusion and PPH in their first birth were at greatest risk of *transfusion* in their second pregnancy (aRR 4.9 (4.1,6.0)), followed by those with transfusion in the absence of PPH diagnosis (aRR 3.2 (2.0,5.3)) and those with PPH alone (aRR 2.2 (2.0,2.5)). The risk of transfusion at the second birth for women with PPH alone was similar to those with morbidly adherent placenta or placenta praevia at their first birth (Table 5).

**Table 4:** Factors associated with increased risk of postpartum haemorrhage in a second birth, amongst women with a first and second birth.

		Postpartum Haemorrhage	aRR^e^	95% Confidence Interval	P value
Private Insurance	Yes	4271/ 87759 (4.9)	0.6	(0.6, 0.6)	< .0001
	No	12034/ 139488 (8.6)	Ref^f^		
Australian born	Yes	11174/ 162402 (6.9)	0.9	(0.9, 0.9)	< .0001
	No	5131/ 64845 (7.9)	Ref		
Smoker	Yes	1623/ 21348 (7.6)	1.0	(0.9, 1.0)	0.2
	No	14638/ 205290 (7.1)	Ref		
Any ART^a^	Yes	559/ 8205 (6.8)	1.2	(1.1, 1.3)	<0.01
	No	15746/ 219042 (7.2)	Ref		
Pregnancy hypertension	Yes	1797/ 24472 (7.3)	1.0	(1.0, 1.0)	0.9
	No	14508/ 202775 (7.2)	Ref		
Chronic hypertension	Yes	117/ 1674 (7.0)	0.9	(0.8, 1.1)	0.5
	No	16188/ 225573 (7.2)	Ref		
Gestational diabetes	Yes	845/ 11646 (7.3)	0.9	(0.9, 1.0)	0.1
	No	15460/ 215601 (7.2)	Ref		
Chronic conditions	Yes	446/ 5610 (8.0)	1.1	(1.0, 1.2)	0.1
	No	15859/ 221637 (7.2)	Ref		
Large for gestational age	Yes	1609/ 17495 (9.2)	1.2	(1.2, 1.3)	< .0001
	No	14696/ 209752 (7.0)	Ref		
Morbidly adherent placenta	Yes	83/ 428 (19.4)	1.7	(1.4, 2.1)	< .0001
	No	16222/ 226819 (7.2)	Ref		
Placenta praevia	Yes	101/ 1644 (6.1)	0.9	(0.7, 1.1)	0.2
	No	16204/ 225603 (7.2)	Ref		
Maternal age	Under 20	1470/ 16631 (8.8)	1.1	(1.1, 1.2)	<0.01
	20–34	13421/ 188582 (7.1)	Ref		
	35+	1413/ 21999 (6.4)	1.0	(0.9, 1.1)	
Socioeconomic status	Highest disadvantage	3431/ 44950 (7.6)	1.1	(1.0, 1.1)	< .0001
	2	3272/ 42651 (7.7)	1.1	(1.1, 1.2)	
	3	3287/ 42426 (7.7)	1.2	(1.1, 1.2)	
	4	3170/ 44902 (7.1)	1.1	(1.0, 1.2)	
	Highest SES^c^	2955/ 50191 (5.9)	Ref		
PPH^b^ Type*	+PPH+RBC^d^	513/ 2399 (21.4)	3.0	(2.8, 3.3)	< .0001
	+PPH -RBC	2806/ 16869 (16.6)	2.4	(2.4, 2.5)	
	-PPH+RBC	58/ 491 (11.8)	1.7	(1.3, 2.2)	
	-PPH-RBC	12928/ 207488 (6.2)	Ref		
Year of birth			1	(1.0, 1.0)	< .0001

^a^ART: Assisted reproductive technology;

^b^PPH: Postpartum haemorrhage;

^c^SES: socioeconomic status;

^d^RBC, Red blood cell;

^**e**^aRR: Adjusted Relative Risk.

^f^Ref: reference category

* +RBC +PPH: Transfusion and haemorrhage; +RBC-PPH: Transfusion without haemorrhage; -RBC +PPH: Haemorrhage without transfusion;-RBC–PPH: neither haemorrhage nor transfusion.

**Table 5:** Factors associated with increased risk of red blood cell transfusion in a second birth, amongst women with a first and second birth.

		Red blood cell transfusion	aRR^e^	95% Confidence Interval	P value
Private Insurance	Yes	549/ 87759 (0.6)	0.6	(0.6, 0.7)	< .0001
	No	1604/ 139488 (1.1)	Ref^f^		
Australian born	Yes	1495/ 162402 (0.9)	0.9	(0.8, 1.0)	0.1
	No	658/ 64845 (1.0)	Ref		
Smoker	Yes	272/ 21348 (1.3)	1.2	(1.0, 1.4)	0.02
	No	1874/ 205290 (0.9)	Ref		
Any ART^a^	Yes	97/ 8205 (1.2)	1.5	(1.2, 1.9)	<0.01
	No	2056/ 219042 (0.9)	Ref		
Pregnancy hypertension	Yes	237/ 24472 (1.0)	1	(0.9, 1.1)	0.8
	No	1916/ 202775 (0.9)	Ref		
Chronic hypertension	Yes	22/ 1674 (1.3)	1.4	(0.9, 2.1)	0.2
	No	2131/ 225573 (0.9)	Ref		
Gestational diabetes	Yes	121/ 11646 (1.0)	1	(0.9, 1.2)	0.7
	No	2032/ 215601 (0.9)	Ref		
Chronic conditions	Yes	60/ 5610 (1.1)	1	(0.8, 1.3)	0.8
	No	2093/ 221637 (0.9)	Ref		
Large for gestational age	Yes	216/ 17495 (1.2)	1.3	(1.1, 1.5)	<0.01
	No	1937/ 209752 (0.9)	Ref		
Morbidly adherent placenta	Yes	15/ 428 (3.5)	2	(1.2, 3.3)	0.05
	No	2138/ 226819 (0.9)	Ref		
Placenta praevia	Yes	37/ 1644 (2.3)	2.3	(1.6, 3.2)	<0.01
	No	2116/ 225603 (0.9)	Ref		
Maternal age	Under 20	235/ 16631 (1.4)	1.3	(1.1, 1.5)	<0.01
	20–34	1715/ 188582 (0.9)	Ref		
	35+	203/ 21999 (0.9)	1.1	(1.0, 1.3)	
Socioeconomic status	Highest disadvantage	530/ 44950 (1.2)	1.4	(1.2, 1.6)	< .0001
	2	460/ 42651 (1.1)	1.3	(1.1, 1.5)	
	3	425/ 42426 (1.0)	1.3	(1.1, 1.5)	
	4	374/ 44902 (0.8)	1.1	(1.0, 1.3)	
	Highest SES^c^	345/ 50191 (0.7)	Ref		
PPH^b^ Type*	+PPH+RBC^d^	111/ 2399 (4.6)	4.9	(4.1, 6.0)	< .0001
	+PPH -RBC	328/ 16869 (1.9)	2.2	(2.0, 2.5)	
	-PPH+RBC	16/ 491 (3.3)	3.2	(2.0, 5.3)	
	-PPH-RBC	1698/ 207488 (0.8)	Ref		
Year of birth			1	(1.0, 1.0)	0.03

^a^ART: Assisted reproductive technology;

^b^PPH: Postpartum haemorrhage;

^c^SES: socioeconomic status;

^d^RBC, Red blood cell;,.

^**e**^aRR: Adjusted Relative Risk.

^f^Ref: reference category

* +RBC +PPH: Transfusion and haemorrhage; +RBC-PPH: Transfusion without haemorrhage; -RBC +PPH: Haemorrhage without transfusion;-RBC–PPH: neither haemorrhage nor transfusion.

## Discussion

Women receiving a blood transfusion in their first birth were less likely to proceed to a second pregnancy and more likely to experience increased morbidity at the second birth than women who did not receive a transfusion. This difference was more pronounced in women who had a transfusion but not a PPH, suggesting an independent effect of transfusion, or another indication for the transfusion.

Women with a first birth transfusion were nearly five times more likely than those without to have a red blood cell transfusion in their second birth. They were also more likely to have a PPH, severe adverse outcome, isoimmunisation or pregnancy loss. The increased risk of a subsequent transfusion found here is similar to that found in a Danish study by Wikkelsø et al, who found a recurrence risk of 4.5 for postpartum transfusion,[7] and a Swedish study by Oberg et al who found recurrence risk of 4.2 for severe PPH (PPH with transfusion or coagulopathy).[6] These studies were both based on births until 2009. In contrast, the recurrence risk of transfusion here is lower than the 11 times recurrence risk of PPH with transfusion found by Ford et al in New South Wales in an earlier period (1994–2002) where transfusion and PPH rates were about half the rates observed in the current study,[5] possibly reflecting a more selective use of transfusion. Although absolute rates of PPH have increased, the approximate 3 times recurrence rate of PPH found in our study is similar to that found elsewhere. [5, 6, 16] This may reflect the smaller proportion of PPH which may result from potentially recurrent causes (retained tissue, coagulation issues) compared with the more common pregnancy specific causes (atony and trauma). However, ascribing PPH cause in these data is difficult and precluded such analysis. [17] Further research into difference in recurrence rates by these sub-types of PPH may be helpful in identifying women at increased need of support in future pregnancies. The higher recurrence rate of PPH with transfusion is not surprising given the previously demonstrated increase in transfusion rates and the known likelihood of recurrent pregnancy complications.

Some previous studies have used blood transfusion as an indicator of severe PPH.[5, 6, 13, 18] In the current study if transfusion were to be considered as a marker of severe PPH, a dose response in adverse outcomes in subsequent pregnancies would be observed. Interestingly those with red blood transfusion in the absence of PPH were the ones at highest risk of adverse outcome in the next pregnancy. These pregnancies are likely to include women with or at risk of antepartum bleeding such as placenta praevia and placental abruption. These conditions are likely to recur in subsequent pregnancies, [19] and have also been associated with subsequent transfusion.[7] These women also had higher rates of use of assisted reproductive technology and pregnancy loss, which may reflect higher rates of pre-existing morbidity in this group. Mothers who were older at their first birth tended to have higher rate of adverse outcomes in their second pregnancy, and younger mothers were at higher risk of haemorrhage or requiring transfusion in their next birth. This increased risk at the extremes of age has been noted in other pregnancy outcomes. [20, 21] The finding of increased risk associated with transfusion without PPH may also reflect poor recording of PPH in the context of factors such as perineal trauma, placental abruption and intrapartum haemorrhage. It was possible that 4% of the women with transfusion without a PPH recorded did actually have a PPH. However, reallocation of these complications as missed PPH diagnoses did not substantially change the results. It is unlikely that all of the women with a transfusion recorded, but no PPH, were missed PPH cases, as their recurrence risk for PPH was substantially lower than their recurrence risk for transfusion.

One concern relating to obstetric blood transfusion is the risk of isoimmunisation in future pregnancies, where the mother produces red cell antibodies from transfusion which can result in haemolytic disease of the newborn in subsequent pregnancies. [22–24] and has been linked to up to 40% of cases of haemolytic disease of the fetus and newborn.[23] Rates of isoimmunisation in this study were in line with the rates of 0.7–1.3% reported elsewhere,[25, 26] with lower rates observed in women not receiving transfusion as expected. Although with routine immunoprophylaxis given to Rh Negative women during pregnancy the risk of isoimmunisation has decreased, [27] the rates of isoimmunisation seen here are a reminder that transfusion is not without risks to future pregnancies.

A large proportion of women receiving a transfusion in the absence of a diagnosis of haemorrhage had hospital records indicating anaemia. Acute anaemia can be the result of haemorrhage and may be treated by transfusion, although in the case of severe haemorrhage, treatment of anaemia following PPH with transfusion has not been shown to meaningfully improve maternal fatigue.[28] Chronic iron deficiency anaemia however, if recognised earlier in pregnancy can be treated by other methods including oral iron and intravenous iron, with national guidelines recommending the use of intravenous iron when a rapid replenishment of iron stores is required. [29]

In the current analysis specifically exploring the impact of first birth factors on second birth severe adverse outcomes, it was women experiencing first birth obstetric transfusion in the absence of postpartum haemorrhage and those experiencing chronic conditions or placenta praevia in their first pregnancy who were most at risk of adverse outcome in the next pregnancy. This highlights the importance of ascertaining first pregnancy complications and potentially discussing appropriate place of birth. Current Australian guidelines recommend that women with identified risk factors for PPH or obstetric haemorrhage be managed throughout pregnancy and birth in such a way as to reduce risks, including consideration of the place of birth and of steps to correct antenatal anaemia. [29, 30] It is encouraging that women who experienced transfusion in their first pregnancy in regional and rural settings tended to birth in higher level care settings for their subsequent birth.

In addition to a pattern of women switching to higher level care following an experience of transfusion in their first birth, there was also a change from public to private care by women who experienced a transfusion and/or a PPH, and a number of women who moved to a lower level of care. This is not surprising, as previous studies have found a link between adverse birth outcomes and changes in care provider for subsequent births.[31] However, this switching between providers is potentially of concern, as there can be differences in the availability and usage of blood products between private and public facilities,[32] and not all hospitals have on-site resources to manage high risk patients.

Overall the median interpregnancy interval was 31 months, which is similar to that reported elsewhere.[31, 33, 34] Women experiencing transfusion, with or without PPH, were less likely to proceed to another pregnancy and when they did they tended to have a longer interpregnancy interval. Reasons for this may be related to the risk factors contributing to the need for transfusion in the initial pregnancy (such as maternal age, anaemia and chronic conditions), or to fear or anxiety related to their first birth experiences. [35, 36] One study which asked women their future pregnancy intentions following a severe PPH in their first birth found that women were less likely to want another pregnancy compared with their pre-pregnancy intentions.[37] In contrast, women in our study experiencing a PPH alone did not have markedly longer interpregnancy intervals than those with no transfusion or PPH.

The strengths of this study included the use of linked population health data to identify births belonging to the same women over time and the large sample size which enabled uncommon outcomes such as transfusion, to be examined. A limitation of this study was the known under-reporting of PPH. However a sensitivity analysis reclassifying likely cases of PPH did not meaningfully change the results. There remains the possibility that some women considered to have experienced transfusion without PPH did actually have a PPH. A further limitation is that there is no information on exact timing of the transfusion, meaning that it is often difficult to determine whether the transfusion occurred before, during or after the birth. Also, indication for transfusion is not recorded in the hospital data. Other risk factors such as obesity and anaemia were not available for all women in the hospital data, with haemoglobin not routinely measured at delivery. Anaemia may contribute to haemorrhage, need for transfusion and morbidity. [38]

## Conclusions

Blood transfusion at a woman’s first birth, particularly in the absence of PPH, was associated with delays in subsequent births, and with higher rates of transfusion and adverse outcomes (including isoimmunisation) at her second birth. While blood transfusion is an appropriate medical treatment for some women, these findings highlight the importance to the clinician of obtaining a complete obstetric history relating to a woman’s history of transfusion and haemorrhage, and using this to ensure the woman is managed in such a way as to minimise her risk in subsequent pregnancies. Despite higher risks in the subsequent births, most women receiving transfusion in their first birth continue on to have uncomplicated second deliveries.

## Supporting information

S1 Fig Study population derivation. Women with first, liveborn singleton deliveries in New South Wales, Australia, 2003–2012.
(TIF)
