# Supplementary figures and images for: Outcomes of subsequent pregnancy following obstetric transfusion in a first birth
Source: PLoS One. 2018 Sep 28;13(9):e0203195. doi: 10.1371/journal.pone.0203195 (PMC6161869; doi:10.1371/journal.pone.0203195)

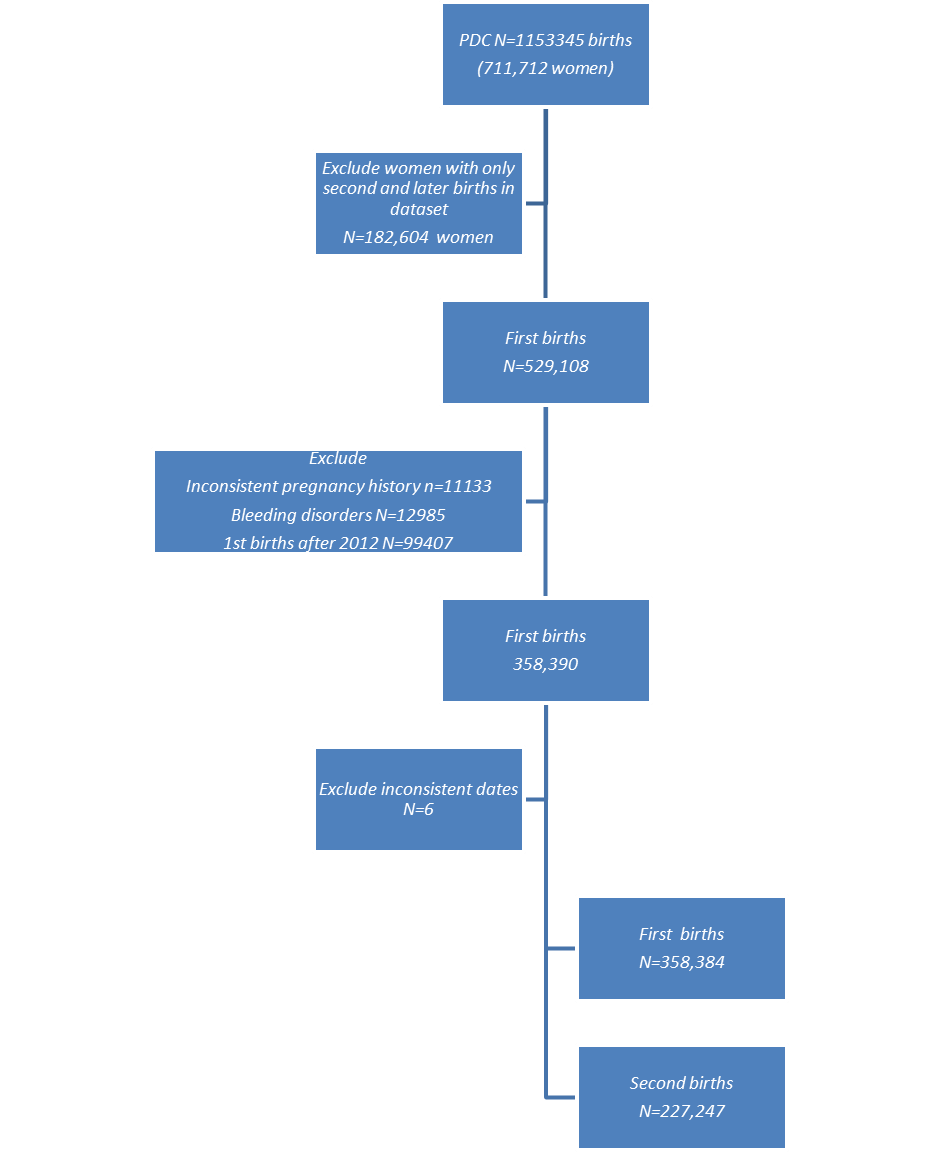

Supplement: S1 Fig — Women with first, liveborn singleton deliveries in New South Wales, Australia, 2003–2012. (TIF) [file pone.0203195.s001.tif]
